# Progress in Surgery

**Published:** 1896-10-03

**Authors:** 


### PROGRESS IN SURGERY.

### SURGERY OF THE NERVES.

**Trifacial Neuralgia.**—The justifiability of the necessarily severe operations undertaken for the relief of this extremely painful affection is gradually being recognised, and the number of patients subjected to the modern operative procedures is undoubtedly on the increase. There are, however, some who hesitate to admit the value of surgical interference in these cases, among them Dr. Gilles de la Tourette,[1] who contributes a paper on the subject from the medical point of view.

In discussing the diagnosis of the condition, he says that the sensory root of the fifth nerve alone is concerned, and he lays particular stress on the "painful spots" where the branches emerge on the face, viz., the supra-orbital, palpebral (at the outer angle of the eye), nasal (at the inner angle), and the ocular points, all associated with the ophthalmic division of the fifth. Those associated with the second or superior maxillary division are the infra-orbital, malar, dental, and palatine; and with the third or inferior maxillary division, the tempero-maxillary, inferior dental, lingual (along the side of the tongue), and mental points. In addition to these points of excessive hyperæsthesia, the whole side of the head, in some cases, is sensitive and tender. In less severe cases only one or two branches may be involved. There are two distinct varieties of trifacial neuralgia. (1) A benign and ephemeral variety, in which the pain is continuous throughout the whole course of the disease, with exacerbations of a shooting character. It is often attributable to external irritation, such as cold, and may follow on influenza or other infective diseases. (2) In true tic douloureaux, on the other hand, the symptoms are always paroxysmal, reaching their acme at once, and subsiding with equal suddenness. In the intervals between the attacks there is complete freedom from pain. The pain is intense while it lasts, and the attacks come on with varying frequency, ten, twenty, or a hundred times a day, but are always of short duration. An attack may be excited by such physiological acts as talking, swallowing, coughing, sneezing, &c., all of which the patient tries to avoid as far as possible. Pressure over the tender spots to some extent relieves the pain, and this being resorted to very frequently may result in producing facial marks, or even deformities.

Vaso-motor and trophic changes often accompany the disease, such as injection of the eyes, œdema of the lids, nasal discharge, excessive salivary secretion, or herpatic eruptions. The essential feature of this variety is its persistence; the older the patient grows the shorter the intervals of freedom from pain become, and the more severe are the attacks.

The etiology of the condition is very obscure. In a few cases the nerve is pressed upon by tumours, syphilitic growths, meningitic patches, or fragments of broken bone, but in the great majority of cases no such definite cause is forthcoming, and the nerve disturbance has to be attributed to such constitutional conditions as gout, rheumatism, diabetes, and so on. Undoubtedly some cases show evidence of being hereditary.

Krause has found pronounced histological changes in the Gasserian ganglion, but none in the peripheral branches of the fifth nerve. Fowler thinks that the sclerotic changes in the vessels may be the essential pathological lesion.

**Differential Diagnosis.**—Typical tic douloureaux has to be diagnosed from (1) the benign form of facial neuralgia; (2) migraine; and (3) hysterical paroxysms in the form of facial neuralgia. These are of long duration, with comparatively long intervals between attacks. The paroxysms come on only two or three times a week, at fixed intervals, last for some hours at a time, and often end with a convulsive seizure and weeping. There is almost always an aura, such as the globus hystericus, noises in the ears, or ephemeral hallucinations. The importance of recognising this clinical form lies in the fact that surgical treatment would be particularly inappropriate. At the same time it is to be borne in mind that true trifacial neuralgia and hysteria may co-exist, as in a case quoted by Tourette.

**Medicinal Treatment.**—The benign variety is always influenced favourably by analgesics—antipyrin, phenacetin, hydrobromate, or valerianate of quinine; whereas true tic defies all such drugs. The writer pins his faith to the treatment advocated by Trousseau and employed by Charcot, namely, the administration of extract of opium in large and progressively increasing doses. He illustrates it by the treatment of a given case. Pills, accurately measured and prepared with a view to ready and complete absorption, were ordered, each containing two centigrammes of the extract of opium. On the first day the patient took three pills at regular intervals, and one additional pill was given each day. When a dose of eight pills had been reached the attacks had decreased by half, and the patient could eat and speak without bringing on a paroxysm. After twelve pills a day the pain had disappeared. For five days this dose was given, and then the pills were reduced at the rate of one every other day, and in twenty-five days the treatment was discontinued. A fortnight later (date of reporting) patient was free from pain and gaining weight.

Unfortunately, this relief can only be looked upon as temporary, and on a further attack supervening the pain is less well borne.

This is the case for medicinal treatment by one of its great supporters, and on such data operative measures are disapproved if not actually condemned as unnecessary.

[1] Med. Week, July 17, 1896.

